# An Australian Neuro-Palliative perspective on Huntington's disease: a case report

**DOI:** 10.1186/s12904-021-00744-z

**Published:** 2021-04-01

**Authors:** Rajvi Shah, Sarah CM Lee, Rupert B Strasser, Christopher Grossman

**Affiliations:** Calvary Health Care Bethlehem, Melbourne, Australia

**Keywords:** Huntington’s disease, Late‐stage, Terminal‐stage, Neuro‐palliative care, Advance care planning, Multidisciplinary team, Advanced

## Abstract

**Background:**

Huntington’s Disease (HD) is an incurable, progressive neuro-degenerative disease. For patients with HD access to palliative care services is limited, with dedicated Neuro-Palliative Care Services rare in Australia. We discuss the experiences of and benefits to a patient with late-stage HD admitted to our Neuro-Palliative Care service.

**Case presentation:**

We present the case of a patient with a 16-year history of HD from time of initial genetic testing to admission to our Neuro-Palliative Care service with late-stage disease.

**Conclusions:**

Given the prolonged, fluctuating and heterogenous HD trajectory, measures need to be implemented to improve earlier access to multi-specialty integrative palliative care services. Given the good outcomes of our case, we strongly advocate for the role of specialised Neuro-Palliative Care services to bridge the gap between clinical need and accessibility.

## Background

Huntington’s Disease (HD) is an incurable, autosomal dominant neurodegenerative disease which causes motor, behavioural and cognitive impairments [1–3]. In recent years, there has been increasing recognition of the role of palliative care in the management of neurodegenerative disease, including motor neuron disease (MND), with emerging literature specific to the need for and benefits of palliative care in advanced HD [4]. Despite this, many mainstream palliative care services do not recognise advanced HD as a terminal illness [5]. We present a case of a patient with late-stage HD admitted to a specialised Neuro-Palliative Care Service, highlighting the critical role of palliative care and the unique challenges associated with its provision in this patient cohort, whilst providing insight into our dedicated multi-disciplinary service model.

## Case Presentation

### Relevant Past History

**Fig. 1:**
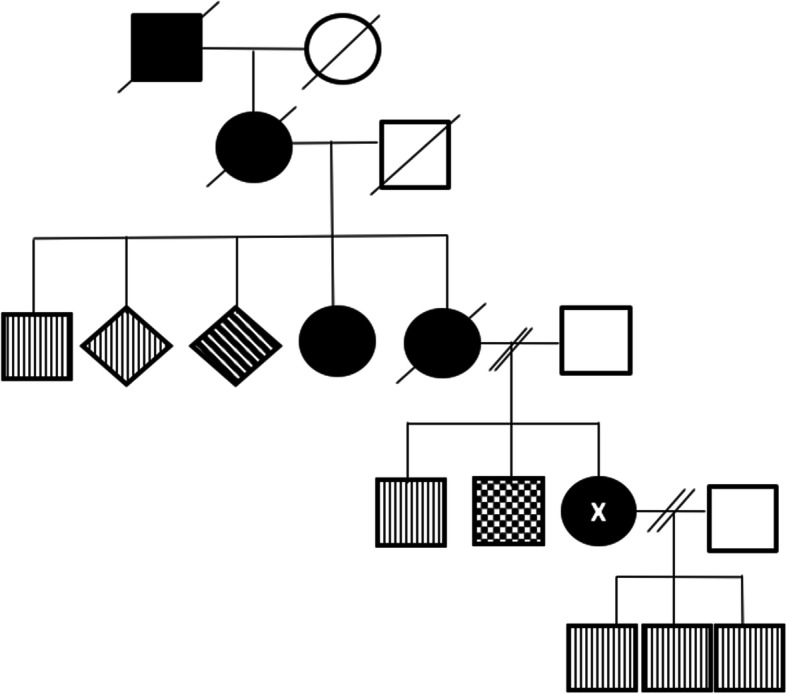
Genogram - Patient denoted by "X". Affected , Gene positive (Asymptomatic) Gene Negative (Asymptomatic) Not Screened (Asymptomatic) Deceased (Asymptomatic)

A 26-year-old woman presented to a presymptomatic genetic testing program to undergo HD gene testing in May 2004. She was married with three sons, aged 2, 4 and 5 and had a strong family history of HD (Fig. 1)*.* Her mother was diagnosed with HD at the age of 33 and died seven years later from the disease. The patient recalled her mother being unprepared for developing HD and reported her desire to complete an Advance Care Plan (ACP) and choose her own Aged Care Facility (ACF) should testing return positive. She denied any symptoms of HD, however physical assessment revealed non-specific motor abnormalities including slurred speech, a shuffling gait with minimal arm swing and gait instability. In July 2004, she was formally diagnosed with symptomatic HD after genetic testing revealed 47 CAG repeats in the huntingtin gene.

From diagnosis until February 2011, she was seen six-monthly at a neuropsychiatry clinic. During this time, she developed anxiety and depression, started using multiple substances (alcohol, marijuana, benzodiazepines and amphetamines) to excess and had three involuntary psychiatry admissions, each after suicide attempts. She was made redundant from her work, divorced and experienced frequent accommodation changes. She also experienced worsening motor and cognitive impairments with consecutive neuropsychiatry assessments demonstrating the emergence of mild executive function deficits, specifically findings of mild reduction in verbal fluency, slowing on tasks of increased cognitive loading and difficulty learning unstructured material. The patient received counselling for poly-substance abuse, eventually abstaining from substance use and was commenced on haloperidol 0.5 mg BD to treat her chorea.

### Referral to our Specialised Neuro-Palliative Care Service

The patient was lost to follow-up until May 2019 when, following re-engagement with community-based HD support groups, she was referred to our Neuro-Palliative Care Service for an outpatient review. The service consists of the State-Wide Progressive Neurological Disease Service (SPNDS), outpatient clinics led by both Neurologists and Palliative Care Physicians and an inpatient ward, where patients are admitted under and primarily managed by a Palliative Care Physician, with a consulting Neurologist, and a community palliative care team. Approximately 50 % of the ward inpatients have a progressive neurological disease, predominantly HD or MND.

The patient had been non-compliant with haloperidol for several years and now had severe chorea with frequent falls. She had deteriorated significantly from a cognitive and functional perspective and had marked weight loss, now weighing 68 kg (previously 120 kg). Formal neuropsychiatry testing was limited by the patient’s inability to grip a pen and form sentences greater than 3 words in length, but revealed severe deficits in verbal skills, visuospatial skills, working memory, new learning and memory, executive function and social cognition consistent with fronto-striatal dementia, typical of advanced HD. Socially, the patient resided in a boarding house, financially supported by a disability pension and had lost contact with her ex-husband and children. Concerns regarding her accommodation suitability, ability to care for herself and medication compliance were raised. She was offered an emergency direct admission to the Neuro-Palliative Ward for symptom assessment and management, which she declined. Haloperidol was re-initiated and due to concerns about the patient’s decision-making capacity an emergency application for medical, lifestyle and financial guardianship was submitted.

### Neuro‐Palliative Ward Admission

In July 2019, following a witnessed fall and paramedic review, the patient agreed to admission to our Neuro-Palliative Ward. Her chorea was managed with a combination of haloperidol 1.5 mg mane and middi and 4.5 mg nocte and tetrabenazine 50 mg TDS. This combination resulted in some motor improvement with an absence of chorea at rest. Only kinesigenic chorea remained, contributing to ongoing high falls risk. Despite the inpatient environment, she continued to have falls during her 5-month long ward admission. Poor visuospatial processing and a busy ward environment precipitated most falls. Some occurred whilst she was in the corridor or near the kitchen attempting to access food. Due to her physical disability the patient previously had limited access to food whilst at the boarding house, resulting in significant meal-time anxiety. A multidisciplinary team (MDT) involving physiotherapy, neuropsychology and nursing staff were involved in addressing her high falls risk and anxiety. Use of mobility aids were limited by her cognitive impairment and injury minimisation strategies with variable patient compliance were implemented, including a low-low bed, soft helmet and hip protectors. To address her anxiety a behavioural support plan was commenced, of which one of the recommendations involved staff members attending the patient’s room 30 min prior to meal delivery to provide reassurance and redirection. This resulted in a reduction in the frequency and severity of falling. Objectively, during the admission, the patient initially had 2–3 unwitnessed falls a week during which she sustained head-strikes, skin lacerations and bruising. Following implementation of the aforementioned measures she had 0–1 witnessed falls a week without associated injuries.

Despite the patient volunteering to complete an ACP, this was not completed at the initial genetic clinic appointment or in subsequent clinic appointments. The patient’s degree of cognitive impairment and lack of family support precluded ACP conversations on admission to the ward, with the lack of an ACP becoming a clinical, legal and ethical issue during the admission. In October 2019, 72-hours after an inpatient fall the patient was noted to have acute deterioration in cognitive processing, prolongation of response latency and further functional deterioration. Given uncertain aetiology of her new symptoms a number of investigations and management courses, including off-site imaging and potential need for surgical intervention, were considered. The treating team opted to clinically monitor her altered conscious state which reverted to her baseline 48-hours later, however continued to fluctuate for the remainder of her ward admission. The treating-team’s opinion was that this was a further manifestation of disease progression into the terminal phase of HD.

In November 2019, members of our MDT attended a suitable ACF prior to the patient’s discharge to perform carer training and education with facility staff, focused on the implementation of the behavioural support plan, harm minimisation strategies and medication management plan. She was discharged with community follow-up by both the SPNDS and community palliative care teams to ensure continuity of care, for further symptom management and eventual provision of end of life care in the ACF.

## Discussion and Conclusions

HD is a chronic and progressive neurodegenerative disease with symptom onset most commonly occurring in the fourth decade of life [1–3]. HD progresses relatively slowly with a majority of patient dying within 10–30 years of diagnosis [6]. HD is caused be an increase in the number of cytosine-adenine-guanine (CAG) repeats in the huntingtin gene [7, 8]. CAG repeats of 39 or greater are associated with symptomatic disease, with greater repeat length correlating with accelerated age of onset and increased disease severity, as occurred in our case [7, 8]. Whilst this gene responsible for HD was identified in 1993, no specific neuroprotective treatment is yet available to prevent the onset in presymptomatic individuals or modify disease trajectory in those who manifest HD [9]. As such, all treatment in HD is tailored to the individual, aimed at maintaining and improving function and quality of life, alleviating symptoms, anticipating clinical and functional problems and ensuring non-abandonment [10, 11]. Comprehensive, multi-disciplinary care is critical for patients with HD and their families at all stages of the disease [12, 13]. At pre-symptomatic, early and middle stages of HD patients benefit from regular follow-up with a range of clinicians (including medical geneticists, neurologists and psychiatrists), assessments and behaviour support strategies by neuropsychologists, rehabilitation and exercise programs, and allied health input including but not limited to physiotherapy, speech therapy training and occupational therapy [14–17]. As HD progresses to the late and terminal stages, collectively known as advanced HD, the need for palliative care services as an adjunct to this multi-disciplinary care, becomes more apparent [18, 19]. We will explore the critical role of palliative care in advanced HD, particularly in late and terminal-stages, highlighting the potential contributions of specialised Neuro-Palliative Care Services to HD care.

To our knowledge, we are the only dedicated Neuro-Palliative Care Service in Australia. Our experience is that patients with HD are reluctant to be admitted to palliative care services. Similarly, palliative care services, including community-based and inpatient consultation and hospice services, are reluctant to admit them. Patient reluctance can stem from beliefs that palliative care is focused solely on death and dying and the age discrepancy between HD patients, who are generally much younger, and the majority of other patients referred to palliative care [2]. Service reluctance is often due to perceptions by staff of people with HD as challenging patients, with difficult to manage behaviours, high psychiatric comorbidity, concerns surrounding legal implications arising from their care (particularly frequent falls and propensity for injury) and prolonged lengths of admission [5, 20]. The unpredictable, idiosyncratic and fluctuating progression of HD over several decades is another barrier [21–23]. Long-term, integrative palliative care required by patients with HD is far removed from the end of life care generally associated with the cancer-care model, and as such palliative care services worldwide are generally not funded nor equipped for the provision of this type of care to these patients [18, 24].

Recognising the limitations of palliative care infrastructure, most literature advocates for the involvement of palliative care in conjunction with existing multi-specialty HD care models once a patient progresses to ‘late-stage’ HD [25, 26]. Late-stage HD is defined as the point at which an individual is no longer able to live independently, requiring twenty-four-hour supervision and assistance with all activities of daily living [25, 26]. Patients typically reach late-stage disease 10–15 years following diagnosis [19]. Signs of terminal-stage include non-ambulatory status, inability or minimal ability to speak or communicate, inability to eat, weight loss, lethargy, excessive sleep or deep lethargy for most of a twenty-four-hour day or conversely, frequent screaming [19, 22, 27]. Terminal HD is regarded as a clinical diagnosis, but biochemical measurements including a serum albumin less than 25 g/L and involuntary weight loss > 10 % are helpful [28]. Some literature suggests lower body mass index and rapid rate of decline can be associated with more rapid disease progression [25]. As both late and terminal-stage HD can be difficult to recognise, involvement of specialised Neuro-Palliative Care services with this expertise can ensure appropriate and timely access to palliative care for HD patients.

To achieve this, our service model involves three distinct neuro-palliative teams, each reflective of HD illness phases: presymptomatic, symptomatic early HD and symptomatic advanced HD, encompassing late and terminal stages. The objectives of care in HD are to ensure quality of life, whilst maximising function and safety, addressing physical and psychiatric manifestations of the disease and providing support to family and care-givers [25, 26]. Our care model enables us to ensure that inpatient admissions may occur when required, that community and end-of-life palliative care support is available and that discussions surrounding ACP can occur earlier in the disease-trajectory. This is highlighted in our case, where our patient was admitted to the unit for symptomatic management and benefitted from the expertise of the MDT in addressing her falls risk, treating chorea and assisting with her discharge planning, including patient-specific carer training and environmental modifications. Further continuity-of-care was arranged via a community link for the patient at the time of discharge.

Palliative care provision for patients with HD is variable and often inadequate. Access to both inpatient and community palliative care remains rare. A recent large Australian population-based study examining the trends in palliative care provision over ten years found that 68.2 % of patients with cancer compared to 45.6 % of patients with MND and 11.6 % of patients with Parkinson’s Disease had access to hospital-based specialist palliative care [24]. Due to low numbers of patients with HD, subgroup analysis for access to palliative care was not possible [24]. As late and terminal stages of HD can extend up to 5 years, with a fluctuating course categorised by periods of deterioration and stability, establishing a link to community palliative care support is difficult [19, 25]. Despite HD patients having a terminal-phase that can last years, Australian patients with HD, on average, are only able to access community palliative care supports 192 days before death [24]. The most common cause of death for HD patients is pneumonia [21–23]. Other common causes of death include other infections, trauma (usually frequent falls), nutritional imbalances, cachexia and cardiovascular disease[21] .

Given the potential causes of death in terminal HD, significant disability, and possibility of invasive life-prolonging interventions, ranging from antibiotics to enteral feeding, clear goals of care are critical [23]. ACP supports an individual alongside their family and health care providers to share their personal values, beliefs, life goals and preferences regarding their care for a future time when they cannot make or communicate their decisions [29]. In patients with HD, ACPs are known to have significant positive impact on their overall quality of life [2, 23, 25, 30]. Despite this, the prevalence of ACPs in HD is low considering the severity, long disease course and its association with premature mortality, with international studies demonstrating only 30–38 % of HD sufferers had completed an ACP [31]. The primary reason for the lack of ACP completion in HD patients appears to be challenges with timing of when to introduce the discussion, similar to the challenges faced with early introduction of palliative care [32]. An understanding of these challenges and the uniqueness of HD patients by Neuro-Palliative care services is core to facilitating ACP completion in this cohort.

As distinct from most patients with cancer, most HD patients and expansion carriers have witnessed a family member suffer from their exact disease and thus know the manifestations and future trajectory of the disease [32]. Additionally, this knowledge and awareness for HD patients typically occurs at a much younger age and earlier disease-stage, often pre-dating disease diagnosis or genetic testing [32]. The period following genetic and clinical diagnosis can be emotionally distressing for patients and their families, marred by shock of a newly diagnosed relative and increased anxiety for at-risk individuals [25].

Following diagnosis, HD patients also face a number of difficult decisions, including planning for the physical effects of deterioration, including choice and location of care and continuation of treatment [25]. A common coping mechanism for the HD family unit is denial; this coupled with the emotional distress, can represent a key barrier to ACP [25]. Furthermore, the unpredictability of disease manifestation and progression means that often an ACP is considered only after HD symptoms have progressed to late-stage disease [2]. As was seen in our patient, by this stage apathy, non-verbal state and cognitive decline often limit the individual’s willingness and ability to engage in ACP conversations [33]. If able to engage, cognitive decline has additional implications on the HD patients’ capacity and decision-making processes [2, 18]. Due to this familiarity with anticipated deterioration, care and symptom needs, as well as establishment of a longitudinal therapeutic relationship with both the patient and their family unit, we believe that involvement of dedicated Neuro-Palliative Care services can have pivotal role in HD care, including facilitation of ACP.

This case report provides insights into a dedicated Neuro-Palliative Care Service and highlights the crucial role palliative care can play in supporting both patients and their families with HD. We advocate strongly that this model of care should be available more broadly. Many current palliative care health models are constrained by a health care system unable to provide the long-term, longitudinal and integrative palliative care HD patients require. To ensure the quality of life of patients with HD is optimised and maintained, in Australia and globally, measures need to be implemented to minimise health inequality and improve HD patients’ access to specialist palliative care services.

## Data Availability

Data sharing is not applicable to this article as no datasets were generated or analysed during the current study.

## Abbreviations

ACF: Aged Care Facility
ACP: Advanced Care Plan/Planning
CAG: Cytosine-adenine-guanine
HD: Huntington’s Disease
MDT: Multidisciplinary team
MND: Motor Neuron Disease
